# Perinatal Use of Melatonin for Offspring Health: Focus on Cardiovascular and Neurological Diseases

**DOI:** 10.3390/ijms20225681

**Published:** 2019-11-13

**Authors:** Chien-Ning Hsu, Li-Tung Huang, You-Lin Tain

**Affiliations:** 1 Department of Pharmacy, Kaohsiung Chang Gung Memorial Hospital, Kaohsiung 833, Taiwan; chien_ning_hsu@hotmail.com; 2 Department of Pediatrics, Kaohsiung Chang Gung Memorial Hospital and Chang Gung University College of Medicine, Kaohsiung 833, Taiwan; litung.huang@gmail.com; 3 Department of Traditional Chinese Medicine, Chang Gung University, Linkow 333, Taiwan

**Keywords:** cardiovascular disease, developmental origins of health and disease (DOHaD), developmental programming, hypertension, melatonin, neurological disease, lactation, oxidative stress, pregnancy

## Abstract

Cardiovascular and neurological diseases can originate in early life. Melatonin, a biologically active substance, acts as a pleiotropic hormone essential for pregnancy and fetal development. Maternal melatonin can easily pass the placenta and provide photoperiodic signals to the fetus. Though melatonin uses in pregnant or lactating women have not yet been recommended, there is a growing body of evidence from animal studies in support of melatonin as a reprogramming strategy to prevent the developmental programming of cardiovascular and neurological diseases. Here, we review several key themes in melatonin use in pregnancy and lactation within offspring health and disease. We have particularly focused on the following areas: the pathophysiological roles of melatonin in pregnancy, lactation, and fetal development; clinical uses of melatonin in fetal and neonatal diseases; experimental evidence supporting melatonin as a reprogramming therapy to prevent cardiovascular and neurological diseases; and reprogramming mechanisms of melatonin within developmental programming. The targeting of melatonin uses in pregnancy and lactation will be valuable in the prevention of various adult chronic diseases in later life, and especially cardiovascular and neurological diseases.

## 1. Introduction

Melatonin, or N-acetyl-5-methoxytryptamine, is a widely distributed molecule in nature. It is ubiquitously present in all living organisms, including microbes, animals, and plants [1,2,3,4]. In animals, melatonin is secreted by the pineal gland from the amino acid tryptophan. Other sources of melatonin include the retina, gut, bone morrow, cerebellum, skin, and a few more. Although melatonin exists widely in many kinds of animal and plant foods, currently melatonin supplementations that are marketed as nutraceutical products are almost all made from synthetic melatonin [2]. 

Chronic diseases such as cardiovascular and neurological diseases are highly prevalent all over the world, despite the advancement in medical treatment [5]. Importantly, most chronic diseases can originate in early life [6]. Environmental insults suffered in utero may increase susceptibility to chronic diseases throughout life. This theory has recently been named the Developmental Origins of Health and Disease (DOHaD) [7]. Conversely, a reprogramming strategy may allow early interventions in the fetal/infantile stage to prevent and reduce long-term negative consequences. It has been reported in the literature that melatonin may be able to reverse the programming process to avoid developing various chronic diseases [8].

Cumulative evidence indicates that melatonin has pleiotropic biological functions which control the circadian rhythm, redox homeostasis, inflammation, energy metabolism, epigenetic regulation, reproductive physiology, and fetal development [1,2,3,4,8,9,10,11,12]. During pregnancy, melatonin produced by the placenta is beneficial to mothers and fetuses [13,14]. Melatonin can pass the placenta and transfer light/dark signals to the fetus [15,16]. During the neonatal period, the production of melatonin by the pineal gland is activated after birth, although it lacks the rhythmic secretion of melatonin until the fetus is 3–5 months old [17]. A growing body of evidence supports the idea that therapeutic use of melatonin during pregnancy and lactation period may reduce materno-fetal complications and prevent neonatal diseases [12,18,19]. 

Currently, melatonin has been used to assist jet lag and sleep disorders as a dietary supplement [20]. In addition, beneficial effects of melatonin have been reported in a variety of diseases [2,4,9], especially with regard to cardiovascular protection or neuroprotection [21,22,23,24]. However, literature focused on the impact of the perinatal use of melatonin within developmental programming of cardiovascular and neurological diseases remains limited. For the purpose of this narrative review, electronic searches were performed in the MEDLINE/PubMed databases. The following keywords were searched: “melatonin”, “phytomelatonin”, “dietary supplement”, “nutraceutical”, “cardiovascular diseases”, “hypertension”, “neurological diseases”, “pregnancy”, “mother”, “maternal”, “gestation”, “lactation”, “neonatal”, “perinatal”, “developmental programming”, “DOHaD”, “offspring”, and “reprogramming”. We reviewed free-access abstracts to determine appropriate studies. English-language published articles were included. Although there were many articles relevant to melatonin in pregnancy and lactation, only a few publications were focused on cardiovascular and neurological outcomes in offspring. 

Here, we review the reported impacts of melatonin uses in pregnancy and lactation and its effects on developmental programming of cardiovascular and neurological diseases. We have particularly focused on the following areas: biofunction of melatonin in pregnancy, lactation, and fetal development; the pathophysiology of melatonin in compromised pregnancy and fetal programming and clinical uses of melatonin in fetal and neonatal diseases; and current evidence of melatonin as a reprogramming therapy in cardiovascular and neurological diseases of developmental origins.

## 2. The Impact of Melatonin in Pregnancy, Lactation, and Fetal Development

### 2.1. Synthesis, Metabolism, and Biofunction of Melatonin

Melatonin is an indoleamine containing a 5-methoxy group and 3-amide group that are added to the indole ring. The biosynthesis of melatonin need four kinds of reaction, including hydroxylation, decarboxylation, acetylation, and methylation. At least six enzymes are involved in the synthesis of melatonin: tryptophan hydroxylase, tryptophan decarboxylase, N-acetylserotonin methyltransferase, tryptamine 5-hydroxylase, serotonin N-acetyltransferase, and caffeic acid O-methyltransferase [25]. Serotonin N-acetyltransferase is the rate-limiting enzyme in melatonin synthesis. Except for tryptophan hydroxylase, all of the above enzymes have been detected in plants [2]. Unlike in animals, melatonin is usually hydroxylated to form 2-, 3-, and 6-hydroxy-melatonin as final products in plants [26]. 

Melatonin is primarily secreted during the night by the pineal gland. Aside from in the pineal gland, melatonin can be produced in many other organs, such as the retina, gastrointestinal tract, skin, lymphocytes, and bone marrow [4]. The half-life of melatonin in the plasma is usually short, being around 20–50 min. In the blood, 70% of plasma melatonin is bound to albumin. Another 30% of melatonin diffuses to nearby tissues [27]. Melatonin is catabolized mainly in the liver by P450 monooxygenases, followed by being conjugated with sulfate to be excreted as 6-sulfatoxy-melatonin in the urine. Additionally, melatonin can be degraded by nonenzymatic pathways. Two melatonin-derived kynuramines of cyclic 3-hydroxymelatonin, N1-acetyl-N2-formyl-5-methoxykynuramine (AFMK) and N1-acetyl-5-methoxykynuramine (AMK) have been discovered [1]. These metabolites of melatonin serve as powerful antioxidants [1,9]. 

Melatonin can regulate a variety of physiological functions in mammals through the activation of two G protein-coupled receptors, the melatonin receptor-1 (MT1) and -2 (MT2). Activation of the MT1 receptor promotes the inhibition of forskolin-stimulated cyclic adenosine monophosphate (cAMP) production [27]. Similarly, the MT2 receptor is also coupled to the inhibition of the forskolin-stimulated cAMP formation [27]. Additionally, melatonin can interact with the nuclear hormone receptor family retinoid Z receptor (RZR)/retinoid acid receptor (ROR) for signal transduction [27]. As a well-known neurohormone secreted from the pineal gland, melatonin has a wide-ranging regulatory and neuroprotective role [23,24]. On the other hand, melatonin controls cardiovascular function by acting either peripherally (e.g., heart and kidney) or centrally (e.g., hypothalamus and rostral ventrolateral medulla) [21,22]. In addition to its role in the cardiovascular and neurological systems, melatonin has multiple receptor-dependent and receptor-independent actions, including having antioxidant and anti-inflammatory properties, being a free radical scavenger, circadian rhythm regulation, cancer inhibition, stimulatory action in mitochondrial biogenesis, immune modulation, blood pressure regulation, and epigenetic regulation [1,2,3,4,9,10,11,12,27,28,29,30]. Notably, melatonin is crucial in pregnancy, delivery, and fetal development [12,13,14,31,32].

### 2.2. Melatonin in Pregnancy and the Fetus

In normal women during pregnancy, nighttime serum melatonin levels are reportedly significantly high throughout pregnancy, reaching a maximum at term and declining to basal levels postpartum [33]. Melatonin can easily cross the placenta and enter the fetal circulation [15]. Photoperiodic information perceived by the mother can thus be transferred to the fetus to synchronize the fetal circadian rhythm via the maternal melatonin rhythm [16]. In pregnant ewes who have received pinealectomy, the daily pattern of fetal breathing movements has showed no rhythm [34], suggesting that photoperiodic information, via maternal melatonin, provides the fetus with light/dark signals. Additionally, melatonin may aid in the delivery of the fetus by enhancing the strength of uterine contractions [35]. Moreover, nocturnal elevation in melatonin level gives rise to increased night-time labor onset and delivery of the offspring [36].

Besides being produced via the pineal gland, the placenta can produce melatonin in a non-circadian fashion and act as an autocrine, paracrine, and endocrine hormone [36]. The placental villous trophoblasts are not only a source of melatonin but also express melatonin receptors [36]. Placenta-derived melatonin can act with the MT1 and MT2 receptors as well as directly scavenge free radicals and reduce oxidative damage to placental tissues. Thus, melatonin in the placenta may protect against oxidative stress attributed to placental dysfunction in compromised pregnancies [29]. Impaired placenta function may cause preeclampsia, leading to oxidative stress. In preeclamptic placentas, the expression of melatonin-synthesizing enzymes, melatonin levels, and melatonin receptor expression are altogether reduced [32]. Conversely, melatonin treatment can prevent preeclamptic sera-induced oxidative stress in the placenta [37]. 

Melatonin is involved in fetal growth and development given the presence of melatonin receptors in several fetal tissues [38]. Studies in rats and mice have shown that melatonin-binding sites in the pituitary gland of the fetus exist as early as day 15 of gestation [39]. Additionally, melatonin receptors are present in many areas of the fetal human brain [40]. Thus, maternal melatonin may play a role in the early stages of fetal development. Since genetic disruption of maternal and embryonic clock function impairs organogenesis in fetus [41], the maternal melatonin rhythm is presumably involved in fetal circadian rhythms and organogenesis. 

Melatonin has been reported to diminish maternal hyperthermia-induced embryo death via restoration of redox balance [42]. Additionally, maternal melatonin can regulate fetal organogenesis that is crucial for successful postnatal adaptation [43]. In a maternal melatonin deficiency rat model, offspring were found to develop disrupted circadian rhythms and intrauterine growth retardation (IUGR), which were prevented by maternal melatonin treatment [44]. These findings support the idea that melatonin acts in different ways in the maternal-placental-fetal system to bring on a successful pregnancy.

### 2.3. Melatonin in Lactation

Upon birth, continuous melatonin provision from the mother and placenta ceases for the newborn. The production of melatonin by the pineal gland is activated after birth. While newborns have a developed pineal gland, they do not produce enough melatonin, especially at night [17]. Thus, young infants do not produce rhythmic melatonin until they are 3–5 months old [17]. In particular, premature infants have been found to have a 2–3-week delay in the development of their melatonin rhythm compared to full-term infants [17]. On the other hand, they have another source of melatonin: breast milk. Since there is a rise in maternal melatonin levels during the night, night-time breastfeeding may cause the transfer of melatonin to the suckling infant [45]. Nevertheless, the role of melatonin addition to a night-time formula feed in infants remains to be determined.

## 3. Clinical Uses of Melatonin

### 3.1. Melatonin Dosage and Side Effects 

In the United Stated and Canada, melatonin is categorized as a dietary supplement, while it is a prescription-only medication in the United Kingdom. Melatonin dosages of up to 10 mg are sold over the counter in Canada and the United States. There are formulations of melatonin in various forms, including tablets, pills, liquids, creams, and sublingual drops. Most formulations come from synthetic melatonin and only a few use plants as a source of melatonin [2]. Additionally, melatonin has been found in all plants and many animal foods. Higher levels of melatonin are noted in eggs and fish than that in meat, while in plant foods, nuts contain the highest melatonin contents, and coffee beans, corn, and medical herbs also have high contents of melatonin [2,20,46].

Oral melatonin supplementation in human studies has ranged from 0.3 mg to 1600 mg daily [47]. Dosages most commonly used are between 2 mg to 10 mg per day across different populations. So far, no serious adverse effects of melatonin in humans have been reported. Fatigue, excessive sleepiness, and reductions in psychomotor and neurocognitive function are the most frequently reported adverse reactions [47]. Despite some animal studies showing that melatonin influences body weight [48,49], a meta-analysis of seven trials recruiting 244 patients did not provide much support for this conclusion [50]. Like adults, melatonin in the pediatric population has a generally favorable safety profile [51,52]. Serious adverse events are scarce in children receiving melatonin treatment [53,54]. Of note is that melatonin appears to elicit pro-inflammatory effects, despite most publications mainly reporting its anti-inflammatory properties [10]. An important focus for research going forward is to simultaneously weight the pro- and anti-inflammatory actions of melatonin in human trials, especially under conditions of autoimmune diseases [10]. 

Currently, no clinical trials of melatonin in pregnant or lactating women have been identified to assess its use and safety in this vital population. Accordingly, due to the lack of human studies, pregnant and breastfeeding women are not recommended to undergo melatonin use [55]. Of note is that when pregnant rats have received high doses of melatonin of up to 200 mg/kg/day during gestational days 6–19 it has seemed not to affect the development of pups adversely [56]. Likewise, when pregnant sheep have received a high dose of melatonin giving rise to concentrations in the range 3–200 times normal concentrations in late gestation there has been no effect on fetal or maternal health, and there has been no effect on myometrial activity [57]. To sum up, melatonin is a relatively safe supplement in humans. However, further research into the long-term offspring outcomes of melatonin use within pregnancy and lactation are urgently required.

### 3.2. Clinical Evidence for Melatonin Use in Fetal and Neonatal Diseases 

As a potential treatment for cardiovascular and neurological diseases, melatonin has been shown to have benefits with regard to hypertension, arrhythmia, myocardial injury, heart failure, pulmonary hypertension, vascular diseases, stroke, Alzheimer’s disease, Parkinson’s disease, brain injury, hypoxia-ischemia encephalopathy, epilepsy, behavior disorders, and sleep disorders as reviewed elsewhere [21,22,23,24]. Because the uses of melatonin in pregnancy and lactation remain inconclusive, we hence only considered works documenting the effects of melatonin from the fetal to the neonatal stage in the current review. Maternal malnutrition and placental dysfunction are the main risk factors of IUGR, which causes premature birth, perinatal death, and a variety of adult diseases like cardiovascular and neurological diseases. Although melatonin has been shown to have a neuroprotective effect in the fetal brain in several animal studies [58,59], little is known about the neuroprotective effect of melatonin use in pregnancy for the human fetus [60].

Table 1 summarizes the main clinical utilities of melatonin for neonatal diseases. Since melatonin easily crosses the blood-brain barrier, it can be administered to reduce the impact of brain lesions in neonates. Perinatal hypoxic-ischemic brain injury is an important problem in the neonates, and leads to cerebral palsy, developmental delay, learning disabilities, and epilepsy. Despite several animal studies having demonstrated that melatonin can reduce oxidative stress induced by hypoxic-ischemic injury and further prevent the ongoing neurological injury process [61,62], only one human trial has supported the conclusion that early administration of melatonin to asphyxiated neonates is able to ameliorate brain injury [63]. Another study has reported that melatonin treatment may be able to reduce mortality in asphyxiated newborns with regard to its antioxidant property [64]. 

Next, respiratory distress syndrome (RDS) and bronchopulmonary dysplasia (BPD) are considered the most common lung diseases among newborns. A previous report has shown that newborns with RDS with higher levels of proinflammatory cytokines and nitrite/nitrate are prone to develop chronic lung disease (CLD) [65]. Because of this finding, Gitto et al. examined the protective effect of melatonin treatment in newborns with RDS grade 3–4 and found that melatonin would lower proinflammatory cytokines and nitrite/nitrate levels and improve the clinical outcome [66]. This group further reported that melatonin treatment protected mechanically ventilated preterm newborns with BPD with regard to its anti-inflammation and antioxidant properties [67].

Because increased oxidative stress is known in septic neonates, melatonin has been examined in newborns with sepsis. In one work, a total of 20 mg melatonin was administered orally in two doses within the first 12 h after diagnosis in 10 septic neonates who showed a significant reduction in levels of lipid peroxidation products and improvement in mortality compared with the non-melatonin treated septic neonates [68]. Additionally, melatonin has been reported to act effectively as a premedication for anesthesia for surgical neonates and as an adjunct analgesic therapy for neonates [69,70]. With regard to the above-mentioned studies, the beneficial effects of melatonin have been mainly attributed to its anti-inflammatory and antioxidant properties. However, it is noteworthy that most studies have had very small sample sizes. Hence, large, prospective, multicenter collaborations are required to conduct meaningful clinical research studies in this specific population to prove that melatonin is an effective therapy in fetal and neonatal disorders. 

## 4. Melatonin Use as a Reprogramming Therapy

### 4.1. Melatonin Therapy in Cardiovascular and Neurological Diseases of Developmental Origins

While current medical treatment for cardiovascular and neurological diseases focuses on high-risk individuals in adulthood [21,22,23,24,71,72], DOHaD concepts offer a ‘reprogramming’ strategy to prevent the development of adult chronic diseases during fetal and infant life [73]. According to the pleiotropically biological functions of melatonin, its use in pregnancy and lactation may reverse adverse programming processes and protect adult offspring against a variety of adult chronic diseases. Although there are several papers relevant to melatonin use in pregnancy and lactation, only small parts of these have been focused on offspring outcomes in adulthood. We only considered works recording outcomes starting from childhood in the current review. The overview of experimental studies in Table 2 shows reports regarding the reprogramming effects of melatonin in a variety of animal studies of programmed cardiovascular and neurological diseases [48,74,75,76,77,78,79,80,81,82,83,84,85,86]. 

Rodents are the dominant animal species used in DOHaD research. Rats reach sexual maturity at approximately 5–6 weeks of age. In adulthood, one rat month is roughly equivalent to three human years [87]. Accordingly, Table 2 lists the ages of reprogramming effects measured in rats as ranging from 11 to 16 weeks, which can be translated to young adult ages in humans. Nevertheless, there is a lack of substantial data regarding the long-term reprogramming effects of melatonin on older adulthood offspring. In addition, limited information is available about the use of large animals in studying the impact of melatonin use in pregnancy and lactation on offspring health.

Early insults that alter in utero development have been linked to adult diseases, including maternal hyperhomocysteinemia [74], maternal caloric restriction [75], N^G^-nitro-L-arginine-methyl ester (L-NAME)-induced preeclampsia [76], maternal high-fructose diet [77], maternal phenytoin exposure [78], maternal continuous light exposure [79,80], maternal high methyl-donor diet [81], maternal high-fructose diet plus post-weaning high-salt diet [82], and glucocorticoid exposure [48,83,84,85,86]. These insults altogether induce adverse cardiovascular and neurological outcomes in adult offspring, including cognition deficits [74,83], neurobehavioral dysfunctions [78,80], and hypertension [48,75,76,77,79,81,82,84,85,86]. All these adverse phenotypes can be prevented, or at least moderated, by melatonin treatment. Of note is that melatonin use in these models of developmental programming is during pregnancy and lactation, which is the developmental stage rather than the established stage of clinical diseases. That is to say, the effects of melatonin on adult offspring are primarily considered to be reprogramming effects instead of direct effects. 

Despite the protective role of melatonin use in pregnancy and lactation having been reported in many models of developmental programming, additional studies are required to clarify the mechanisms driving reprogramming effects, appropriate therapeutic windows for melatonin administration, and ideal doses and timing before clinical translation.

### 4.2. Reprogramming Effects of Melatonin on Developmental Programming

**Figure 1:**
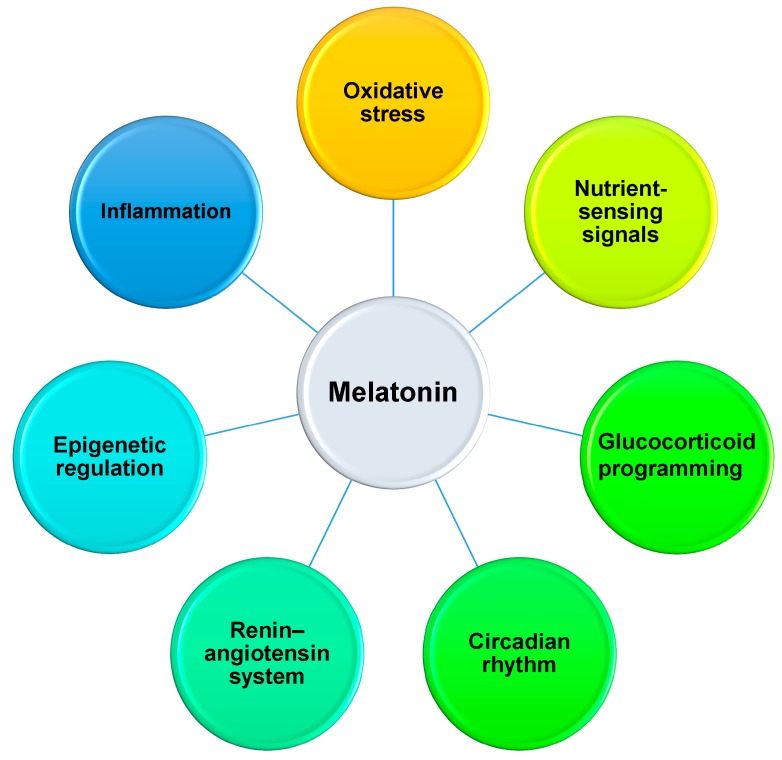
Schema outlining the potential mechanisms that may underlie the reprogramming effects of melatonin uses in pregnancy and lactation to prevent the developmental programming of cardiovascular and neurological diseases in later life.

Despite the common mechanisms underpinning developmental programming remaining elusive, emerging evidence from animal studies has afforded insight into pathways, including oxidative stress [88,89], renin-angiotensin system (RAS) [90], nutrient-sensing signaling [89,91], inflammation [10], epigenetic gene regulation [92,93,94], circadian rhythm [95], and glucocorticoid programming [96]. Notably, extensive experimental animal studies have demonstrated interplay between melatonin and the above-mentioned mechanisms [8,28]. Figure 1 is a graphic illustration of the reprogramming mechanisms of melatonin interrelated to developmental programming of adult diseases.

The fetus has low-antioxidant capacity which is not sufficient to overcome reactive oxygen species (ROS) overproduction in response to adverse environments in utero. Thus, oxidative stress may cause harm to the developing fetus [88]. It is well known that melatonin acts as an antioxidant for protection against oxidative stress [1]. Not only melatonin but a series of its metabolites act as antioxidants [1,9]. As we have reviewed elsewhere [8,89], numerous early-life insults have been reported to cause developmental programming which is linked to oxidative stress, including maternal undernutrition, maternal overnutrition, maternal diabetes, preeclampsia, prenatal hypoxia, maternal exposure to nicotine or ethanol, maternal inflammation, glucocorticoid exposure, and maternal high methyl-donor diet. Among these, beneficial effects of maternal melatonin therapy have been shown in models of maternal caloric restriction [75], L-NAME-induced preeclampsia [76], maternal high-fructose diet [77], maternal high methyl-donor diet [81], and glucocorticoid exposure [83]. Since nitric oxide (NO) is a key mediator of blood pressure regulation and NO deficiency is a common mechanism underlying programmed hypertension [97], melatonin use in pregnancy and lactation may have beneficial effects via restoration of the NO-ROS balance in a variety of hypertension models of developmental programming [75,76,77,82]. These observations support the idea that melatonin works as an antioxidant in different ways to prevent adult diseases of developmental origin. 

Secondly, melatonin is involved in epigenetic regulation [10,28]. Epigenetic mechanisms such as posttranslational modification of histones, DNA methylation, and RNA interference play central roles in gene regulation [98]. In a prenatal dexamethasone exposure model [83], melatonin was shown to protect against alterations of hippocampal morphology and restore reelin mRNA expression levels by reducing DNMT1 expression. Additionally, melatonin was seen to reduce reelin expression via disassociation of DNMT1 and methyl-CpG binding protein 2 (MeCP2) from its promoter. Furthermore, melatonin and trichostatin A (a histone deacetylase (HDAC) inhibitor) have similar protective effects on neonatal dexamethasone-induced programmed hypertension [84], suggesting that melatonin might act like a HDAC inhibitor to epigenetically regulated hypertension-related genes to prevent programmed hypertension. Interestingly, we have analyzed the renal transcriptome of male offspring exposed to melatonin during pregnancy and lactation at three different ages [28]. Using RNA next-generation sequencing, 455, 230, and 132 differentially expressed genes were identified at 1, 12, and 16 weeks of age, respectively. Maternal melatonin therapy up-regulates rather than down-regulates genes in the offspring kidney. Our findings are consistent with previous studies which have reported that melatonin can act like an inhibitor of DNA methyltransferases (DNMT) or serve as an HDAC inhibitor [10,84]. Moreover, several epigenetic regulator genes have been observed to be up-regulated during kidney development [28]. Hence, the epigenetic mechanisms of melatonin in developmental programming deserve additional research.

Thirdly are studies of crosstalk between melatonin and the RAS. The RAS is a well-known hormonal cascade controlling kidney development and blood pressure [99]. In melatonin-deficient hypertension [100], the classical RAS, defined as the angiotensin-converting enzyme (ACE)/angiotensin (Ang) II/angiotensin type 1 receptor (AT1R) axis, is activated. Under suboptimal in utero conditions, environmental stimuli activate the classical RAS axis, resulting in renal programming and consequent hypertension in later life [11]. Conversely, melatonin uses in pregnancy and lactation block the activation of the RAS and prevent the development of hypertension in various animal models, including maternal caloric restriction [75], maternal L-NAME exposure [76], maternal high-fructose diet [77], maternal continuous light exposure [79,80], and glucocorticoid exposure [48,84]. Apart from the classical RAS axis, the impact of the ACE2–angiotensin (1–7)–Mas receptor axis has also been studied in developmental programming [101]. So far, studies have produced conflicting results with up- and down-regulation of different components of the intrarenal RAS being reported [102]. In general, RAS expression is reduced at birth and becomes normalized with age. Nevertheless, this normalization may be inappropriately high in adulthood, and, hence, activate the classical RAS during kidney development [102]. Although early blockade of the classical RAS between 2–4 weeks of age has been reported to prevent programmed hypertension [103,104], it is still unclear how and when to target which RAS element(s) to avoid the programming of cardiovascular and neurological diseases. A previous study demonstrated that maternal melatonin therapy induces mRNA expression of *Agtr1b* and *Mas1* to prevent programmed hypertension in a prenatal dexamethasone plus post-weaning high-fat diet model [86]. These findings suggest that RAS may be an important mechanism contributing to reprogramming effects of melatonin and the developmental programming of hypertension. Nevertheless, whether there is any inappropriate RAS activation during kidney development leading to negative consequences caused by perinatal use of melatonin warrants further investigation.

Nutrient-sensing signaling is another potential mechanism relevant to reprogramming effects of melatonin able to prevent the development programming of disease. Several forms of nutrient-sensing signaling are involved in developmental programming, such as silent information regulator T1 (SIRT1), AMP-activated protein kinase (AMPK), peroxisome proliferator-activated receptors (PPARs), and PPARγ co-activator 1α (PGC-1α) [89,91]. Maternal malnutrition and metabolic dysfunction can disrupt nutrient-sensing signaling, which is a major determinant of fetal metabolism and development [89,94,105]. Early-life nutritional and metabolic insults impair nutrient-sensing signaling to mediate PPARs and their target genes, thereby promoting programmed hypertension [91]. Hypertension programmed by a maternal methyl-donor diet is related to decreases in several forms of nutrient-sensing signaling, including *Sirt1, Prkaa2, Pparb,* and *Pparg* [76]. Another study demonstrated that maternal melatonin therapy prevented a hypertension programmed high-fructose/high-salt diet by regulating *Sirt1, Sirt4, Prkaa2, Prkab2, Pparg,* and *Ppargc1a* [77]. Importantly, activation of the AMPK/SIRT1/PGC-1α pathway has been shown to reverse the programming process and prevent hypertension [106]. Therefore, results from these studies suggest a close link among nutrient-sensing signaling, circadian rhythm, and melatonin in the developmental programming of adult disease.

Moreover, neuroinflammation is a characteristic of several neurodegenerative diseases, including Alzheimer’s disease, amyotrophic lateral sclerosis, Parkinson’s disease, Huntington’s disease, spinocerebellar ataxia, and multiple sclerosis [107]. Suppression of endogenous melatonin synthesis by inflammation has been reported in multiple sclerosis, an autoimmune inflammatory disease [108]. Conversely, emerging evidence supports the modulatory effects of melatonin in several inflammatory diseases [10,109]. Anti-inflammatory actions of melatonin are related to SIRT1 [107]. These include nuclear factor erythroid 2-related factor 2 (NRF2) activation and inhibition of NF-*κ*B activation and the NOD-like receptor family, pyrin domain-containing 3 (NLRP3) inflammasome [107]. Additional studies are required to develop prenatal inflammation models to examine the interrelationships between maternal inflammation, melatonin signaling, and neurodevelopmental programming underlying these findings.

Lastly, emerging evidence supports the interplay between glucocorticoid and melatonin within developmental programming. Exposure of a developing fetus to excessive glucocorticoids can occur via a number of mechanisms, such as exogenous administration, preterm birth, and stressed pregnancies. High glucocorticoid exposure in early life can adversely program the fetal hypothalamic–pituitary–adrenal (HPA) axis and increase susceptibility to developing various adult chronic diseases [96]. Conversely, melatonin therapy has positive effects on cognition deficits [83] and hypertension [48] programmed by prenatal dexamethasone. In addition, hypertension programmed by neonatal glucocorticoid exposure in adult offspring can be prevented by perinatal melatonin uses [85]. Circulating glucocorticoid levels have been recognized as a major internal synchronizer of the circadian system. Melatonin can down-regulate glucocorticoid receptor expression [110] while MT receptors may be down-regulated following dexamethasone treatment [85]. Therefore, these findings indicate a possible crosstalk between melatonin and glucocorticoid by which both chronobiotics tightly mediate developmental programming processes. Like melatonin, breast-milk glucocorticoids might also influence the development of circadian rhythms in infants, although this has not been studied thus far. 

## 5. Conclusions

Melatonin acts in a variety of ways to have impacts on human health. There is general agreement that melatonin therapy is well tolerated and has a favorable safety profile across different populations. During pregnancy, melatonin can transfer photoperiod information from mother to fetus and is involved in fetal development. In the maternal pinealectomy-induced melatonin-deficient model, adult offspring have been seen to develop a variety of adverse outcomes in later life. Conversely, emerging evidence from animal models of developmental programming suggests that melatonin uses in pregnancy and lactation may serve as a reprogramming strategy against the development of cardiovascular and neurological diseases. Although there have been clinical trials aiming to figure out the ideal therapeutic period and dose of melatonin with which to treat neonatal and fetal diseases, the therapeutic use of melatonin during pregnancy and lactation as a reprogramming therapy for various adult chronic diseases, especially cardiovascular and neurological diseases, still awaits further clinical translation. 

There are several limitations to our review. The beneficial effects of perinatal melatonin administration are attributed to several mechanisms that are known to interrelate with the pleiotropic functions of melatonin, but on the other hand, these biofunctions might cause negative effects. Maternal melatonin is able to cross the placenta and enter the fetal circulation, this being another form of action which is called the transgenerational effect [111]. Considering this, additional studies are needed to clarify whether there is any negative consequence caused by its perinatal use on their offspring. In addition, we observed that follow-up periods after the cessation of melatonin use were relatively short in almost all animal studies. Future studies should determine the long-term reprogramming effects of melatonin as a dietary supplement or as a nutraceutical in humans. With a better understanding of the ideal treatment duration and dosage of melatonin in pregnancy and lactation, we shall be able to promote better maternal and child health, and especially cardiovascular and neurological outcomes.

## Figures and Tables

**Table 1:** Clinical applications of melatonin in neonatal diseases.

Clinical Condition	Study Design	Main Results	References
Perinatal hypoxic-ischemic	Thirty newborns with hypoxic-ischemic encephalopathy received enteral dose of melatonin 10 mg/kg daily for five days	Reduced mortality and improved brain injury	[63]
Perinatal hypoxic-ischemic	Ten asphyxiated newborns received a total of 80 mg of melatonin (eight doses of 10 mg each separated by 2 hr intervals) orally.	Reduced mortality	[64]
Respiratory distress syndrome	Sixty newborns received 10 intravenous injections of melatonin (10 mg/kg each)	Reduced proinflammatory cytokines	[65]
Respiratory distress syndrome grade 3–4	Twenty-four newborns received 10 intravenous injections of melatonin (10 mg/kg each)	Reduced proinflammatory cytokines and improved outcome	[66]
Bronchopulmonary dysplasia with ventilator	Fifty-five preterm newborns received 10 intravenous injections of melatonin (10 mg/kg each)	Reduced proinflammatory cytokines and improved outcome	[67]
Sepsis	Ten septic newborns received a total of 20 mg oral melatonin in two doses of 10 mg each, with a 1 h interval.	Reduced mortality	[68]
Surgery	Five newborns received a total of 10 doses of melatonin (10 mg/kg) 3 h after the end of surgery.	Reduced proinflammatory cytokines and nitrate/nitrite levels	[69]
Adjunct analgesic therapy	Thirty preterm newborns received 10 mg/kg of intravenous melatonin prior to intubation	Reduced pain score and proinflammatory cytokines	[70]

Studies tabulated according to clinical condition.

**Table 2:** Reprogramming effects of melatonin in animal models of developmental programming.

Dose and Period of Melatonin Treatment	Animal Models	Species/Gender	Age at Evaluation	Reprogramming Effects	References
Melatonin 10 mg/kg/day s.c. throughout pregnancy	Maternal methionine intake-induced hyperhomocysteinemia	Wistar rat/M and F	75 days	Prevented cognition deficit	[74]
0.01% melatonin in drinking water during pregnancy and lactation	Maternal caloric restriction	SD rat/M	12 wks	Prevented hypertension and increased renal NO	[75]
0.01% melatonin in drinking water during pregnancy and lactation	Maternal L-NAME exposure	SD rat/M	12 wks	Prevented hypertension and increased renal NO	[76]
0.01% melatonin in drinking water during pregnancy and lactation	Maternal high-fructose diet	SD rat/M	12 wks	Prevented hypertension and increased renal NO	[77]
Melatonin (40 μg/mL) in drinking water from gestational days 0 to 19	Maternal phenytoin exposure	Wistar rat/M and F	12 wks	Protected neurobehavioral dysfunctions	[78]
0.01% melatonin in drinking water during pregnancy and lactation	Maternal continuous light exposure	SD rat/M	12 wks	Prevented hypertension	[79]
Melatonin 1 mg/kg s.c. injection at circadian time 12, from day 17 to 21 of pregnancy	Constant light exposure from gestational day 10 to 21	Wistar rat/M	16 wks	Protected anxiety-like and sexual behaviors	[80]
0.01% melatonin in drinking water during pregnancy and lactation	Maternal high methyl-donor diet	SD rat/M	12 wks	Attenuated hypertension and altered renal transcriptome	[81]
0.01% melatonin in drinking water during pregnancy and lactation	Maternal high-fructose diet plus post-weaning high-salt diet	SD rat/M	12 wks	Attenuated hypertension and restored NO system	[82]
0.01% melatonin in drinking water during pregnancy and lactation	Prenatal dexamethasone exposure	SD rat/M	16 wks	Protected hippocampal morphology and reelin level	[83]
0.01% melatonin in drinking water during pregnancy and lactation	Prenatal dexamethasone exposure	SD rat/M	16 wks	Prevented hypertension and increased nephron number	[48]
0.01% melatonin in drinking water during pregnancy and lactation	Neonatal dexamethasone exposure	SD rat/M	16 wks	Prevented hypertension and preserved histone deacetylase gene expression	[84]
0.01% melatonin in drinking water during lactation	Neonatal dexamethasone exposure	SD rat/M	16 wks	Prevented hypertension and increased renal melatonin level and MT2 protein	[85]
0.01% melatonin in drinking water during pregnancy and lactation	Prenatal dexamethasone exposure plus post-weaning high-fat diet	SD rat/M	16 wks	Prevented hypertension and up-regulated *Agtr1b* and *Mas1* expression	[86]

Studies tabulated according to animal models, species, and age at evaluation. Legend: SD, Sprague-Dawley; M, male; F, female; s.c., subcutaneous; L-NAME, N^G^-nitro-l-arginine methyl ester.

## Abbreviations

ACE	Angiotensin-converting enzyme
AFMK	N1-acetyl-N2-formyl-5-methoxykynuramine
AMK	N1-acetyl-5-methoxykynuramine
AMPK	AMP-activated protein kinase
AT1R	Angiotensin type 1 receptor
BPD	Bronchopulmonary dysplasia
CLD	Chronic lung disease
DNMT	DNA methyltransferases
DOHaD	Developmental origins of health and disease
HDAC	Histone deacetylase
HPA	Hypothalamic–pituitary–adrenal
IUGR	Intrauterine growth retardation
L-NAME	N^G^-nitro-L-arginine-methyl ester
MT	Melatonin receptor
NLRP3	NOD-like receptor family, pyrin domain-containing 3
NRF2	Nuclear factor erythroid 2-related factor 2
PPAR	Peroxisome proliferator-activated receptors
RAS	Renin–angiotensin system
RDS	Respiratory distress syndrome
ROS	Reactive oxygen species
SD	Sprague-Dawley
SIRT1	Silent information regulator T1
